# Effect of dexamethasone and ramosetron on the prevention of postoperative nausea and vomiting in low-risk patients: a randomized, double-blind, placebo-controlled, multicenter trial

**DOI:** 10.1186/s12871-023-02334-3

**Published:** 2023-11-07

**Authors:** Jong-Ho Kim, Jin-Sun Kim, Yeong-Gwan Jeon, Jangho Bae, Kiyoung Shin, Byeongmun Hwang

**Affiliations:** 1 Department of Anesthesiology and Pain Medicine, Hallym University Chuncheon Sacred Heart Hospital, Hallym University Medical Center, 77 Sakju-ro, Chuncheon, 24253 Republic of Korea; 2 Department of Anesthesiology and Pain Medicine, Gangneung Asan Hospital, College of Medicine, University of Ulsan, Gangwon, Republic of Korea; 3 Department of Anesthesiology and Pain Medicine, Wonju College of Medicine, Yonsei University Hospital, Wonju, Republic of Korea; 4 Department of Anesthesiology and Pain Medicine, School of Medicine, Kangwon National University Hospital, Kangwon National University, Chuncheon, 24341 Gangwon-do Republic of Korea

**Keywords:** Antiemetics, Dexamethasone, General anesthesia, Postoperative nausea and vomiting, Prophylaxis, Ramosetron

## Abstract

**Background:**

Several studies have investigated the effect of antiemetics on postoperative nausea and vomiting (PONV) in high-risk groups. However, few studies have investigated the effect of antiemetics in patients at low risk of developing PONV.

**Methods:**

In this prospective, randomized, double-blinded trial, 177 patients undergoing surgery under general anesthesia were randomly allocated to three groups. Patients allocated to group C (control group) received 2 mL of intravenous 0.9% saline, those allocated to group R (ramosetron group) received 0.3 mg of intravenous ramosetron, and those allocated to group DR (ramosetron plus dexamethasone group) received 5 mg of intravenous dexamethasone and 0.3 mg of intravenous ramosetron.

**Results:**

Finally, 174 patients completed the study, and the types of surgeries were orthopedic (n = 80), rhinologic (n = 47), urologic (n = 29), and others (n = 18). The incidence of PONV up to 48 h postoperatively was significantly lower in group DR than in group C. The incidence of PONV up to 0–1 h postoperatively was significantly lower in groups R and DR than in group C. The usage pattern of rescue antiemetics was consistent with the incidence of PONV. The percentage of patients requiring rescue analgesics 0–1 h postoperatively was significantly lower in groups R and DR than in group C.

**Conclusions:**

The combination of dexamethasone and ramosetron demonstrated a superior effect in preventing PONV for 48 h after surgery under general anesthesia than saline in patients at low risk of developing PONV. Compared with saline injections, ramosetron injections yielded better outcomes for the incidence of PONV and the use of rescue antiemetics and rescue analgesics 0–1 h postoperatively.

**Trial registration:**

Clinical trial registration number: criskorea@korea.kr, KCT0006749.

## Introduction

Postoperative nausea and vomiting (PONV), a frequent complication of general anesthesia, has an overall incidence of 40–90% [1]. PONV is generally self-limiting; however, rare but serious medical complications, such as aspiration of gastric contents, esophageal rupture, suture dehiscence, subcutaneous emphysema, and pneumothorax, may result from PONV [2]. These complications delay recovery and discharge and can increase overall healthcare costs significantly [3]. Thus, various pharmacological agents, such as anticholinergics, antihistamines, promethazine, neurokinin-1 inhibitors, corticosteroids, and 5-hydroxytryptamine type 3 (5-HT_3_) receptor antagonists, have been used for the prevention and treatment of PONV [4, 5].

Selective 5-HT_3_ receptor antagonists are widely used as first- and second-line drugs to prevent PONV owing to their efficacy and limited side effects [6, 7]. The antiemetic effect of ramosetron is more potent and longer than that of previous 5-HT_3_ receptor antagonists owing to its strong binding affinity and slower dissociation from 5-HT_3_ receptors [8]. Dexamethasone, another antiemetic, has proven efficacy in reducing PONV [9]. The combination of dexamethasone and ramosetron, a commonly evaluated combination of antiemetics, reportedly enhances antiemetic efficacy compared with 5-HT_3_ antagonists alone [5, 10].

Several studies have evaluated the effect of antiemetics on PONV in high-risk groups [2–6]. However, the effect of antiemetics on PONV in low-risk groups has not been clarified. The prediction of PONV is inaccurate according to known predictive models, and the incidence of PONV may be high even in low-risk patients [4, 5, 7]. Therefore, analyzing the effect of prophylactic administration of antiemetics on nausea and vomiting for PONV in low-risk groups is essential. To the best of our knowledge, no previous study has investigated the effect of ramosetron and dexamethasone on the prevention of PONV in low-risk patients. We hypothesized that the incidence of PONV would be lower in patients who received ramosetron and dexamethasone than in those who received saline. Therefore, in this prospective, randomized, double-blind, placebo-controlled study, we aimed to investigate the effect of ramosetron and dexamethasone on the prevention of PONV in patients at low risk of developing PONV.

## Materials and methods

This prospective, randomized, multicenter, double-blinded trial enrolled patients scheduled to undergo surgery under general anesthesia at university hospitals. The patients were recruited between January 2022 and December 2022. All patients were expected to receive intraoperative antiemetics or saline. The study design was approved by the Institutional Review Boards of the Hallym University Chuncheon Sacred Heart Hospital (IRB no. 2021-11-002), Wonju Severance Christian Hospital (IRB no. CR121091), Gangneung Asan Hospital (IRB no. GNAH 2022-01-002), and Kangwon National University Hospital (IRB no. 2021-09-014). Written informed consent was obtained from each participant before the administration of any study drug. This trial was registered at the Clinical Research Information Service (cris.nih.go.kr, 11/11/2021, KCT0006749) and conducted in accordance with the Declaration of Helsinki.

The participants were adults aged ≥ 20 years scheduled to undergo surgery under general anesthesia classified as American Society of Anesthesiologists physical status I or II and determined to have two or fewer risk factors for PONV. The risk of PONV was calculated based on six risk factors: female sex, surgery with a high risk of nausea and vomiting, history of PONV, non-smokers, younger age (age < 50 years), and postoperative use of opioid analgesics [4]. The exclusion criteria included the following: use of drugs, such as narcotic analgesics, antiemetics, and steroids, within 24 h before surgery; patients with a history of allergy to dexamethasone or ramosetron; patients with symptoms of vestibular dysfunction; and patients undergoing surgeries associated with a high risk of nausea and vomiting, such as laparoscopic surgery, gallbladder surgery, and gynecological surgery. Patients were enrolled and divided into control (C), ramosetron (R), and dexamethasone-ramosetron (DR) groups, with each group comprising 59 patients.

The computer-generated random allocation sequence was created by an independent investigator with a 1:1:1 allocation and random block sizes; the group assignment was prepared by the enrolling anesthesiologist in sealed opaque envelopes. The enrolling anesthesiologist was not the same person as the treating anesthesiologist. The envelopes were opened before induction of anesthesia, and the drugs were prepared by an independent nurse who was not participating in any other part of the study. Participants and outcome assessors were blinded to group allocation. Patients in group C received intravenous saline (2 mL) at the end of the surgery, those in group R received intravenous ramosetron (0.3 mg; 2 mL) (Nasea®, Daiichisankyo.co.kr, Seoul, Republic of Korea) at the end of the surgery, and those in group DR received a combination of intravenous dexamethasone (5 mg; 1 mL) before the induction of anesthesia and intravenous ramosetron (0.3 mg; 2 mL) at the end of the surgery [9, 11].

All patients were allowed to consume solid food for up to 8 h before the surgery and water for 2 h before the surgery. Standard monitoring, including limb lead electrocardiography, pulse oximetry, noninvasive blood pressure measurements, end-tidal anesthetic gas concentration measurements, and capnography, was performed when the patient arrived in the operating room. Anesthesia was induced by administering 1.5–2.0 mg/kg of propofol and 0.5–0.8 mg/kg of rocuronium bromide intravenously. Endotracheal intubation was performed following 2–3 min of mask ventilation with 100% oxygen. Anesthesia was maintained with 1.5–3% sevoflurane and air in oxygen (fraction of inspired oxygen: 0.5). The administration of sevoflurane was discontinued, and neuromuscular blockade was antagonized via the intravenous administration of a combination of 0.3 mg/kg of pyridostigmine and 0.008 mg/kg of glycopyrrolate at the end of the surgery. Extubation was performed when the patients fully regained consciousness. All participants received no opioids during the operation.

The demographic data, duration of anesthesia, and duration of surgery were recorded for each patient. All episodes of PONV were recorded during the first 48 h following emergence from general anesthesia at the following time intervals: 0–1, 1–6, 6–24, and 24–48 h. Nausea was defined as a subjectively disagreeable sensation accompanying the urge to vomit, and vomiting was defined as the forceful ejection of gastric contents from the mouth.

The severity of nausea was assessed using an 11-point verbal numerical rating scale (VNRS), with the scores ranging from 0 to 10, where a score of 0 indicates no nausea and a score of 10 indicates the worst nausea imaginable. Based on the VNRS scores, the severity of nausea was classified as mild (1–3), moderate (4–6), and severe (7–10). These assessments were performed in conjunction with the PONV assessments. The rescue antiemetic, 10 mg of metoclopramide or 0.3 mg of ramosetron, was administered intravenously for the treatment of severe nausea or two or more emetic episodes or upon request from the patient. If PONV persisted after the administration of the rescue antiemetic, 4 mg of ondansetron was administered intravenously. The frequency of rescue antiemetic administration was also recorded. The patients were instructed to rate the severity of pain during the 48-h postoperative study period using an 11-point VNRS similar to that used in the assessment of the severity of nausea. An intravenous bolus dose of 30 mg of ketorolac or 20 mg of nefopam was administered upon request from the patient or when the VNRS pain score was ≥ 5. The frequency of rescue analgesic administration was recorded. In addition, data regarding the incidence of adverse effects, such as dizziness, headache, and drowsiness, were also collected. All data were recorded independently by an anesthesiologist who was blinded to the group allocation of the patients.

## Outcomes

The primary outcome measure of this study was the incidence of PONV during the first 48 h after the surgery. The secondary outcome measures were the use of postoperative rescue antiemetics and rescue analgesics.

### Sample size and statistical analyses

Because the incidence of PONV in low-risk patients has not been reported previously, we conducted the preliminary study. In a preliminary study, 20 patients (with the same inclusion and exclusion criteria) were administered intravenous saline (2 mL) at the end of the surgery, and the incidence of PONV was 35%. The data from the preliminary study were not included in the present study. A sample size calculation was performed using a power analysis (α = 0.05, 1-β = 0.9). A 25% difference in the incidence of PONV for the saline group versus the dexamethasone-ramosetron group indicated that 54 patients per group were required. Assuming a potential dropout rate of 10%, the final sample size was set at 177 patients. The data are expressed as the mean ± standard deviation (SD) or number (%) of patients.

For intergroup comparisons, the normality of the distribution of continuous variables was assessed using the Levene test. Normally distributed data are presented as the mean ± SD and analyzed using one-way analysis of variance. Categorical variables were analyzed using the chi-squared test or Fisher’s exact test, as appropriate. All significant results were further analyzed using Scheffe’s post-hoc test to detect intergroup differences. The *P*-values were adjusted using the Bonferroni correction if a statistically significant difference was noted in multiple comparisons among the groups. Two-sided *P*-values < 0.05 were considered statistically significant. SPSS software (version 25.0; IBM Corp., Armonk, NY, USA) was used to analyze the data.

## Results

**Fig. 1:**
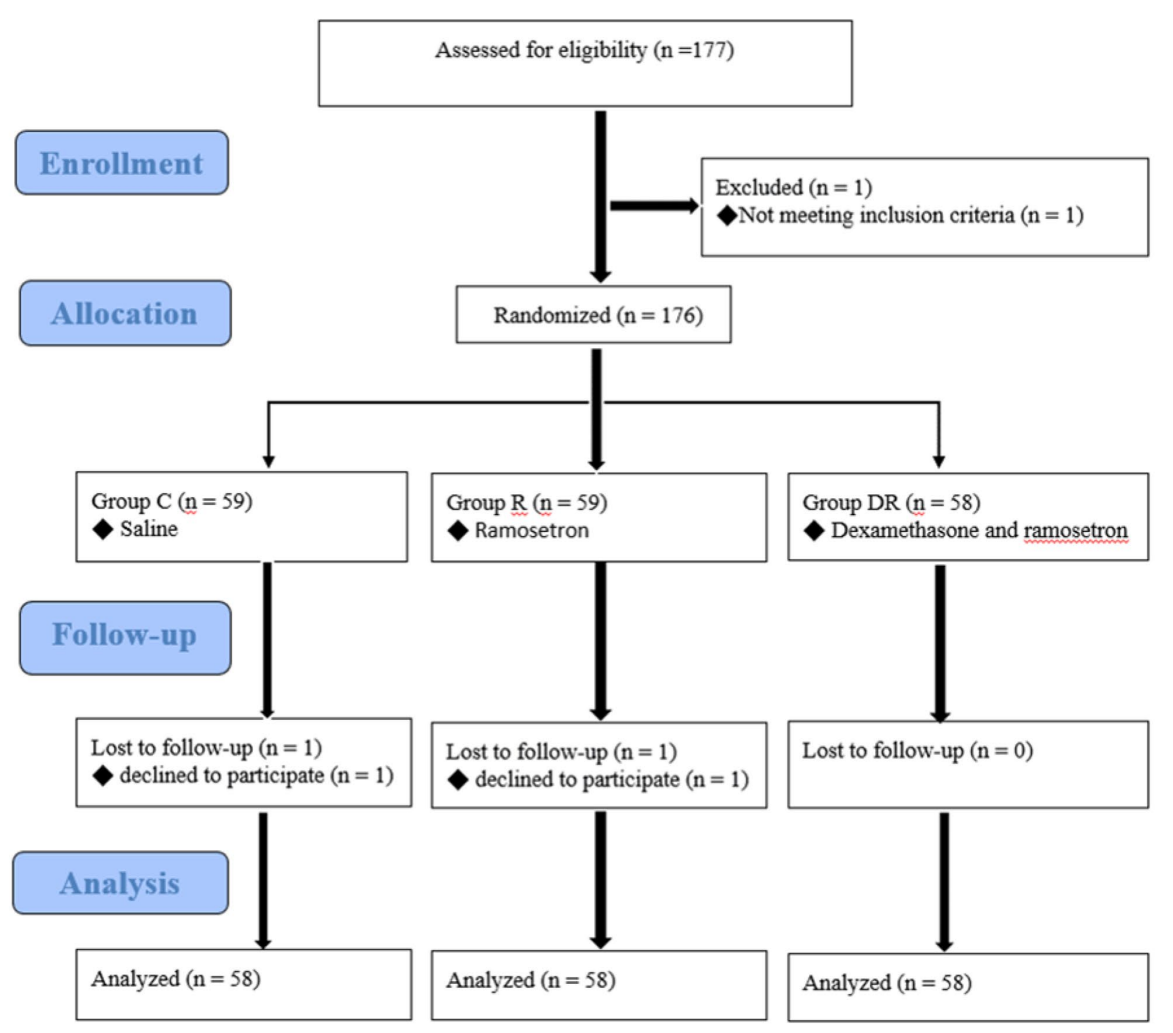
CONSORT diagram of the study design

A total of 177 patients were randomized after providing consent (Fig. 1). One patient in group C and one patient in group R withdrew from the study after randomization for personal reasons. One patient in group DR was excluded as he did not meet the inclusion criteria. Thus, 174 patients completed the trial. Patient characteristics, including age, sex, weight, height, smoking status, Apfel score, duration of surgery, duration of anesthesia, American Society of Anesthesiologists physical status classification, and type of surgery, did not differ among the three groups (*P* > 0.05; Table 1). The data of continuous variables were normally distributed.

**Table 1:** Baseline demographic and clinical characteristics of the patients

Characteristic	Group C (n = 58)	Group R (n = 58 )	Group DR (n = 58)	*P value*
Age (years)	56.5 ± 10.0	59.2 ± 8.3	56.4 ± 11.7	0.247
Sex (female)	6 (10%)	7 (12%)	10 (17%)	0.521
Weight (kg)	71.0 ± 10.3	69.8 ± 11.3	69.9 ± 12.9	0.612
Height (cm)	166.9 ± 7.3	167.7 ± 6.1	167.3 ± 8.2	0.603
Nonsmoking status	26 (45%)	33 (57%)	35 (60%)	0.212
Apfel score, n, (%)				0.835
0 1 2	10 (17%) 25 (43%) 23 (40%)	14 (24%) 21 (36%) 23 (40%)	13 (22%) 20 (34%) 25 (43%)	
Duration of surgery (min) Duration of anesthesia (min) ASA status I/II, n, (%)	85.3 ± 61.5 122.6 ± 67.0 30 (52%)/28 (48%)	73.0 ± 50.5 110.4 ± 62.1 30 (52%)/28 (48%)	61.9 ± 38.2 97.1 ± 48.6 39 (67%)/19 (33%)	0.691 0.713 0.150
Type of surgery				0.862
Orthopedic surgery	23 (40%)	29 (50%)	28 (48%)	
Rhinologic surgery	16 (27%)	14 (24%)	17 (29%)	
Urologic surgery	11 (19%)	10 (17%)	8 (14%)	
Other	8 (14%)	5 (9%)	5 (9%)	

Data are presented as mean score ± SD or n (%) of patients. Data were analyzed using analysis of variance (continuous variables) or the χ^2^ test (incidence variables). Patients in Group C received 2 mL of intravenous 0.9% saline at the end of surgery; those in group R received 0.3 mg of intravenous ramosetron at the end of surgery; and those in group DR received 5 mg of intravenous dexamethasone at the induction of anesthesia and 0.3 mg of intravenous ramosetron at the end of surgery. The Apfel risk score consists of four predictors: nonsmoking, female sex, history of motion sickness and/or postoperative nausea and vomiting, and postoperative opioid usage. ASA: American Society of Anesthesiologists physical status

Cumulative results for the 0–48 h study period revealed that the overall incidence of nausea was significantly lower in group DR than in group C (*P* < 0.05; Table 2); however, no significant difference was observed between groups C and R. The incidence of nausea was the highest during the 0–1 h study period, which subsequently decreased throughout the study period. Nausea was reported in 15 (26%) patients in group C, nine (16%) in group R, and five (9%) in group DR 48 h after the surgery. None of the patients in this study experienced postoperative vomiting. Table 2 presents the number of patients in each group who experienced at least one episode of nausea within the four time intervals. The incidence of nausea at the four intervals, except for the first 1 h after the surgery, did not differ significantly among groups C, R, and DR: 14 (24%), five (9%), and four (7%) patients, respectively, 1 h after the surgery; six (10%), three (5%), and three (5%) patients, respectively, from 1 to 6 h after the surgery; three (5%), four (7%), and two (3%) patients, respectively, from 6 to 24 h after the surgery; and four (7%), three (5%), and two (3%) patients, respectively, from 24 to 48 h after the surgery. The incidence of nausea during the first 1 h after the surgery was significantly lower in groups R and DR than in group C; in contrast, no significant difference was observed between groups R and DR. No significant differences were observed in the severity of nausea at 0–1, 1–6, 6–24, and 24–48 h postoperatively among the three groups. Administration of rescue antiemetic drugs was required in 14 (24%) patients in group C, seven (12%) in group R, and five (9%) in group DR during the 48-h study period. The overall frequency of antiemetic rescue drug use was significantly lower in group DR than in group C. The frequency of rescue antiemetic drug use during 1–6 h was significantly lower in groups R and DR than in group C; however, no significant difference was observed between groups R and DR. No significant differences were observed in the number of patients requiring rescue antiemetics during 1–6, 6–24, and 24–48 h postoperatively among the three groups (*P* > 0.05 for all comparisons; Table 2).

**Table 2:** Incidence of postoperative nausea and vomiting and use of rescue antiemetics

Characteristic	Group C (n = 58)	Group R (n = 58)	Group DR (n = 58)	*P value*
Postoperative 0–1 h				
Incidence of nausea Severity of nausea (0/1/2/3) Rescue antiemetic usage	14(24%) 44/8/4/2 12 (21%)	5(9%)^*^ 53/2/3/0 4 (7%)^*^	4(7%)^*^ 54/1/2/1 4 (7%)^*^	0.010 0.069 0.027
Postoperative 1–6 h				
Incidence of nausea Severity of nausea (0/1/2/3) Rescue antiemetic usage	6 (10%) 42/5/1/0 6(10%)	3 (5%) 55/1/2/0 2 (3%)	3 (5%) 55/2/1/0 3 (5%)	0.447 0.284 0.283
Postoperative 6–24 h				
Incidence of nausea Severity of nausea (0/1/2/3) Rescue antiemetic usage	3 (5%) 55/3/0/0 3 (5%)	4 (7%) 54/3/0/1 3 (5%)	2 (3%) 56/1/1/0 2 (3%)	0.704 0.521 0.877
Postoperative 24–48 h				
Incidence of nausea Severity of nausea (0/1/2/3) Rescue antiemetic usage	4 (7%) 54/4/0/0 4(7%)	3 (5%) 55/2/1/0 3 (5%)	2 (3%) 56/1/1/0 2 (3%)	0.704 0.552 0.704
Postoperative 0–48 h				
Incidence of nausea Severity of nausea (0/1/2/3) Rescue antiemetic usage	15 (26%) 43/9/4/2 14 (24%)	9 (16%) 49/4/4/1 7 (12%)	5 (9%)^*^ 53/2/2/1 5 (9%)^*^	0.043 0.273 0.048

Data are presented as n (%) of patients. Data were analyzed using analysis of variance (continuous variables) or the χ^2^ test (incidence variables). Patients in group C received 2 mL of intravenous 0.9% saline at the end of surgery; those in group R received 0.3 mg of intravenous ramosetron at the end of the surgery; and those in group DR received 5 mg of intravenous dexamethasone at the induction of anesthesia and 0.3 mg of intravenous ramosetron at the end of surgery. Nausea: 0, none; 1, mild; 2, moderate; 3, severe; emetic episode, retching or vomiting. None of the patients experienced postoperative vomiting. ^*^P < 0.05 in comparison with group C (The *p****-***values are based on Scheffe’s post-hoc test adjusted using the Bonferroni correction)

The pain intensity scores at each time interval and percentages of patients who received rescue analgesics at 0–1, 1–6, 6–24, and 24–48 h did not differ among the groups (Table 3). The percentages of patients who required the administration of rescue analgesics during 0–1 h postoperatively were significantly lower in groups R (22/58, 38%) and DR (22/58, 38%) than in group C (40/58, 69%; *P* < 0.05; Table 3).

**Table 3:** VNRS scores for pain and rescue analgesic data up to 48 h after anesthesia

Characteristic	Group C (n = 58)	Group R (n = 58 )	Group DR (n = 58)	*P value*
VNRS score for pain				
Postoperative 0–1 h Postoperative 1–6 h Postoperative 6–24 h Postoperative 24–48 h	2.8 ± 1.8 2.6 ± 1.7 1.7 ± 1.5 1.3 ± 1.0	2.7 ± 1.5 2.2 ± 1.6 1.3 ± 1.2 0.7 ± 1.1	2.4 ± 1.5 1.9 ± 1.2 1.4 ± 1.0 1.0 ± 0.8	0.618 0.595 0.609 0.197
Rescue analgesic				
Postoperative 0–1 h Postoperative 1–6 h Postoperative 6–24 h Postoperative 24–48 h	40 (69%) 25 (43%) 23 (40%) 14 (24%)	22 (38%)^*^ 16 (28%) 24 (41%) 7 (12%)	22 (38%)^*^ 15 (26%) 24 (41%) 6 (10%)	0.001 0.091 0.976 0.082

Data are presented as mean score ± SD or n (%) of patients. Data were analyzed using analysis of variance (continuous variables) or the χ2 test (incidence variables). Patients in group C received 2 mL of intravenous 0.9% saline at the end of surgery; those in group R received 0.3 mg of intravenous ramosetron at the end of surgery; and those in group DR received 5 mg of intravenous dexamethasone at the induction of anesthesia and 0.3 mg of intravenous ramosetron at the end of surgery. The verbal numerical rating scale (VNRS) assigned scores from 0–10, with 0 indicating no pain and 10 indicating the worst pain possible. ^*^P < 0.05 in comparison with group C (The *p****-***values are based on Scheffe’s post-hoc test adjusted using the Bonferroni correction)

The adverse effects observed in groups C, R, and D were dizziness (none [0%], three [5%], and one [2%], respectively), headache (five [9%], three [5%], and one [2%], respectively), and drowsiness (none in all three groups). No significant differences were observed among the three groups in terms of the incidence of these adverse effects, which were relatively mild in all groups (*P* > 0.05; Table 4).

**Table 4:** Incidence of adverse events

Adverse effects	Group C (n = 58)	Group R (n = 58 )	Group DR (n = 58)	*P value*
Dizziness Headache Drowsiness	0 (0%) 5 (9%) 0 (0%)	3 (5%) 3 (5%) 0 (0%)	1 (2%) 1 (2%) 0 (0%)	0.167 0.245 0

Data presented as n (%) of patients. Patients in group C received 2 mL of intravenous 0.9% saline at the end of surgery; those in group R received 0.3 mg of intravenous ramosetron at the end of surgery, and those in group DR received 5 mg of intravenous dexamethasone at the induction of anesthesia and 0.3 mg of intravenous ramosetron at the end of surgery

## Discussion

This is the first randomized, double-blind study to investigate the effect of ramosetron and dexamethasone on the prevention of PONV in patients at low risk of developing PONV after surgery under general anesthesia. Compared with group C, group DR showed a lower incidence of PONV and a reduction in the use of rescue antiemetics and rescue analgesics for 48 h postoperatively in low-risk patients undergoing surgery under general anesthesia. Moreover, compared with group C, group R showed a lower incidence of PONV and a reduction in the use of rescue antiemetics and rescue analgesics 0–1 h postoperatively.

Selective serotonin 5-HT3 receptor antagonists, such as ramosetron, have a well-established role in the prophylaxis and treatment of PONV owing to their efficacy and fewer side effects compared with other antiemetics [7, 8]. Dexamethasone, a glucocorticoid, also produces antiemetic effects, possibly by releasing endorphins and inhibiting prostaglandin and serotonin production [9, 12]. A meta-analysis showed that a combination of dexamethasone and a 5-HT3 receptor antagonist, such as ramosetron, is more effective in preventing PONV than 5-HT3 antagonists alone, and the need for rescue antiemetic was reduced in patients receiving this combination [13]. However, the results of individual studies differ. In patients undergoing thyroid surgery, the incidence of nausea and need for rescue antiemetics were significantly lower in groups DR and R than in group C. Compared with ramosetron alone, the combination of ramosetron and dexamethasone significantly reduced the incidence of nausea and the need for rescue antiemetics [14]. However, Jeon et al. reported that the incidence of PONV, the severity of nausea, and the need for rescue antiemetics did not differ significantly between patients receiving a combination of ramosetron and dexamethasone and those receiving ramosetron alone for the prevention of PONV after thyroidectomy [15]. In patients undergoing laparoscopic cholecystectomy, the combined use of ramosetron and dexamethasone was more effective in reducing the need for rescue antiemetics and severity of nausea than ramosetron alone; however, it did not reduce the overall incidence of PONV [16]. Yang et al. reported that in highly susceptible patients undergoing spinal surgery, the combination of ramosetron and dexamethasone significantly reduced the incidence of moderate-to-severe nausea and vomiting than ramosetron alone; however, the incidence of total PONV was similar between the groups [10]. The results of these studies are inconsistent with those of previous studies in which dexamethasone combined with another 5-HT3 antagonist (ondansetron) decreased the incidence of PONV [17, 18].

Previous studies focused on the prevention of PONV in high-risk groups; however, this study focused on a low-risk group and showed that the incidence of nausea and need for rescue antiemetics 0–1 h postoperatively was significantly lower in groups DR and R than in group C. In addition, the incidence of nausea and need for rescue antiemetics were significantly lower in group DR than in group C 48 h postoperatively. However, the incidence of nausea and the need for rescue antiemetics did not differ significantly between groups DR and R. The incidence of PONV after laparoscopic surgery in high-risk patients ranges from 40 to 80% [13]. In this study, the incidence of PONV after surgery under general anesthesia in low-risk patients was 17% (control group, 26%). The lack of a difference between groups R and DR in this study may be attributed to the low incidence of PONV.

In this study, patients underwent surgeries under general anesthesia with sevoflurane and had a low risk of developing PONV (Apfel score, 1.2 ± 0.7). The incidence of nausea 1 and 48 h postoperatively was 9% and 16%, respectively, when 0.3 mg of ramosetron was administered at the end of the surgery, and the need for rescue antiemetics 1 and 48 h postoperatively was 7% and 12%, respectively. Yoo et al. reported the administration of 0.3 mg of ramosetron to patients at low risk of developing PONV (Apfel score, 1.7 ± 0.5) who underwent laparoscopic radical prostatectomy and general anesthesia with desflurane [19]. In their study, the incidence of nausea 1 and 48 h postoperatively was 22.6% and 58.1%, respectively, and the need for rescue antiemetics 1 and 48 h postoperatively was 12.9% and 22.6%, respectively. The higher incidence of PONV may be attributed to the type of surgery (robot-assisted laparoscopic radical prostatectomy) and the exclusion of surgical factors from the Apfel score. Kim et al. reported the administration of 0.3 mg of ramosetron to patients who underwent gynecological laparoscopic surgery under general anesthesia (Apfel score, 3.7) [20]. In their study, the incidence of nausea at 0–2 and 48 h postoperatively was 9.1% and 34.1%, respectively, and the need for rescue antiemetics at 0–2 and 48 h postoperatively was 11.4% and 20.5%, respectively. Thus, the incidence of PONV and need for rescue antiemetics in this study were lower than the corresponding findings in previous studies [19, 20], indicating that the effect of ramosetron on the incidence of nausea and need for rescue antiemetics is evident in high-risk as well as low-risk patients with low Apfel scores.

Single-dose dexamethasone is effective for the management of postoperative pain after various surgeries, including laparoscopic surgery [21, 22]. The possible mechanisms underlying the analgesic effect of single-dose dexamethasone include anti-inflammatory activity and the modulation of systemic physiological responses [23]. Lee et al. reported that pain scores and rescue analgesic consumption were significantly lower in groups R and DR than in group C 1 h after thyroid surgery [14]. However, in this study, the pain scores of the three groups were similar 0–1 h postoperatively (mean score, 2.6), and the percentage of patients requiring rescue analgesics 0–1 h postoperatively was significantly lower in groups R and DR than that in group C. These results may indicate that ramosetron and dexamethasone are effective in reducing severe pain immediately after surgery. The percentages of patients requiring rescue analgesics 1–6, 6–24, and 24–48 h postoperatively were similar among the groups, which may be attributed to the study including patients undergoing surgeries that cause less pain.

We monitored the patients for major adverse effects related to the administration of ramosetron and dexamethasone, such as headache, dizziness, and drowsiness, for 48 h postoperatively. Both drugs were tolerated well during the study period, and the addition of dexamethasone to ramosetron did not influence the incidence of adverse events. The incidence of these adverse effects (8%) was lower than that of PONV (17%). The incidence of side effects, such as headache, dizziness, and drowsiness, was lower than that reported in previous studies (25–39%) [14, 15]. The prolonged use of steroids, such as dexamethasone, is associated with dangerous adverse effects, including an increased risk of wound infection and anastomotic leak [23]. However, adverse effects, such as wound infection or anastomotic leaks, were not observed in any of the patients in this study. Thus, a single dose of dexamethasone does not appear to increase the incidence of these adverse effects [24].

This study has a limitation. Our study included patients who had undergone several types of surgery. However, only patients with relatively short surgical times were studied to minimize the confounding effects of surgery.

## Conclusions

The combination of dexamethasone and ramosetron was superior to saline in preventing PONV for 48 h after surgery under general anesthesia in patients at low risk of developing PONV. Compared with saline, administration of ramosetron alone was associated with a lower incidence of PONV and a reduction in the use of rescue antiemetics and rescue analgesics 0–1 h postoperatively.

## Data Availability

The datasets generated during and/or analyzed during the current study are available from the corresponding author on reasonable request.

## Abbreviations

PONV: postoperative nausea and vomiting
C: control
R: ramosetron
DR: dexamethasone-ramosetron
